# Vaginal Hysterectomy with Anterior Four-Arm Mesh Implant Technique in the Surgical Treatment of a Woman with Total Pelvic Organ Prolapse and Urinary Incontinence: A Case Report and Review of the Literature

**DOI:** 10.1155/2016/2906596

**Published:** 2016-08-29

**Authors:** Gökmen Sukgen, Esra Saygılı Yılmaz, Eralp Başer

**Affiliations:** ^1^Department of Gynecology and Obstetrics, Metro Hospital, 06520 Adana, Turkey; ^2^Department of Gynecology and Obstetrics, Adana Numune Training and Research Hospital, 06520 Adana, Turkey

## Abstract

*Purpose*. We present a case report of a woman with total POP and SUI who was treated with a technique utilizing vaginal hysterectomy followed by the placement of a four-arm synthetic polypropylene mesh implant system.* Methods*. An 81-year-old grand-multiparous woman presented to our clinic complaining of a vaginally protruding mass and urinary incontinence. A surgical approach including vaginal hysterectomy, anterior four-arm mesh implant, posterior large segment vaginal enterocele repair, and perineoplasty with levator ani fixation was planned.* Results*. The patient was discharged home at the second postoperative day. Follow-up visits at the first, 3rd, and 6th months were normal. There was complete symptomatic relief and objective cure of the POP and urinary incontinence symptoms.* Conclusion*. We believe that anterior four-arm mesh implant and large posterior repair should be considered after vaginal hysterectomy. Future studies are needed to evaluate the utility of this technique for treatment of POP.

## 1. Introduction

Pelvic organ prolapse (POP) is defined as the prolapse of the pelvic organs toward or through the vaginal opening as a result of the weakening of the combination of nerves, muscles, and fascia, which normally protect and support the physical position of pelvic organs [1, 2]. Of all the women with stress urinary incontinence (SUI), 63% also have prolapse; and the treatment of symptomatic POP can only be achieved with surgery.

Herein, we present a case report of a woman with total POP and SUI who was treated with a technique utilizing vaginal hysterectomy followed by the placement of a four-arm synthetic polypropylene mesh implant system, which enables fixation in anterior prolapse from 4 points, as an alternative to the conventional surgery (vaginal hysterectomy and cystorectocele repair). The technique presented enhances anatomical and functional recovery and also provides support as a tension free midurethral sling for the treatment of urinary incontinence.

## 2. Case Presentation

**Figure 1:**
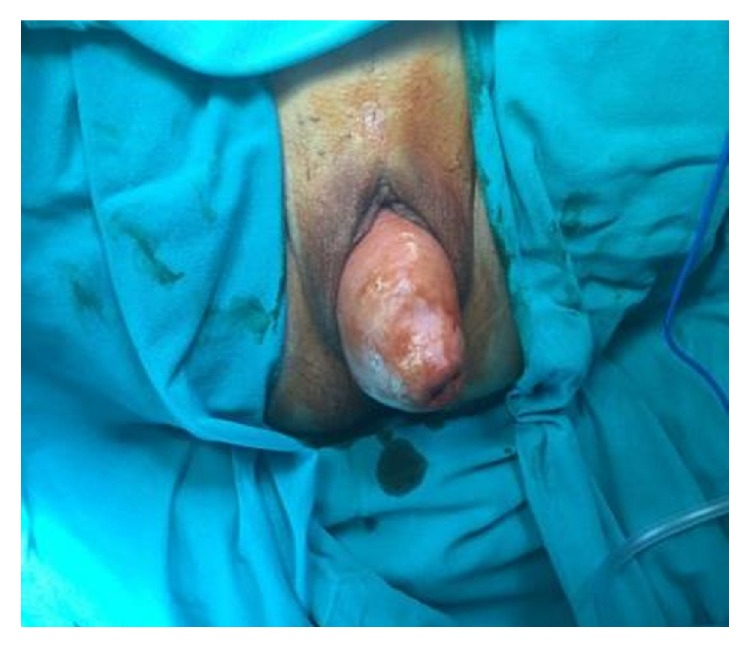
Preoperative findings of the patient with total pelvic organ prolapse with anterior-posterior and apical prolapse (grade IV).

An 81-year-old grand-multiparous woman (G10 P8) presented to our clinic complaining of a vaginally protruding mass and urinary incontinence. The urinary incontinence was mixed (urgency and stress incontinence); however, the SUI component was dominant. Her past medical history was unremarkable. The general systemic physical examination was normal. On pelvic examination, total pelvic organ prolapse with anterior-posterior and apical prolapse (grade IV) was noted (Figure 1). A surgical approach including vaginal hysterectomy, anterior four-arm mesh implant, posterior large segment vaginal enterocele repair, and perineoplasty with levator ani fixation was planned.

**Figure 2:**
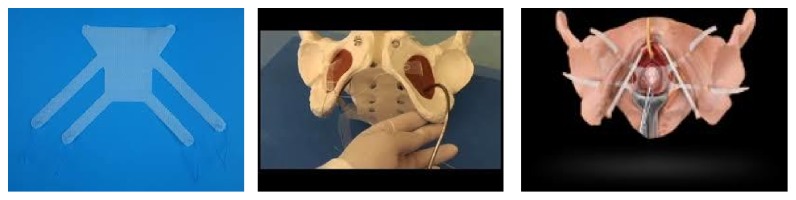
The posterior part of the four-arm mesh was passed through the obturator foramen.

**Figure 3:**
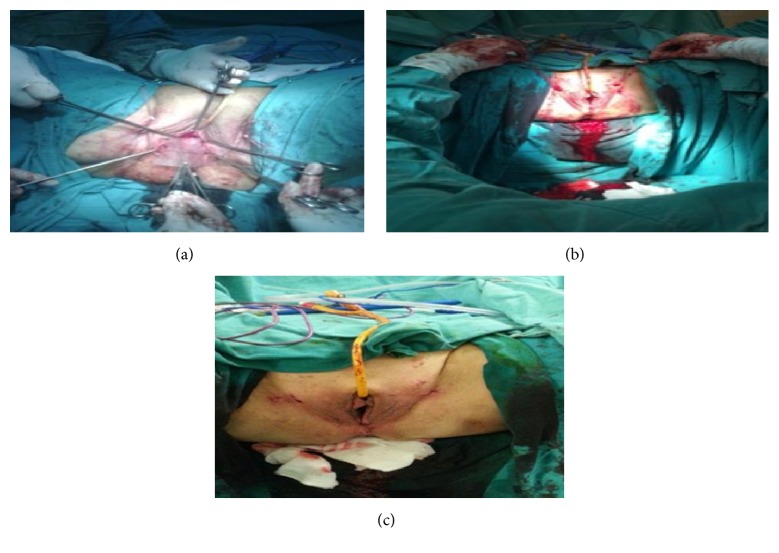
(a) shows that the anterior arms were placed as a midurethral sling as in the intravaginal slingplasty (IVS) procedure, supporting the midurethra and bladder. (b) shows that mesh was fixed using tension free technique. (c) shows postoperative findings of the patient.

On the operation day, the patient was placed in dorsal lithotomy position under spinal anesthesia. A pericervical circular incision was performed on the vaginal mucosa. The vesicovaginal cleavage plane was found, and the bladder was retracted anteriorly. From the posterior of the cervix, peritoneal entry into posterior cul-de-sac was accomplished. Bilateral cardinal ligaments and sacrouterine ligaments were clamped with Heaney clamps, cut, and suture ligated. Bilateral uterine arteries were clamped, cut, and suture ligated. The anterior cul-de-sac peritoneum was opened. The uterine fundus was held with clamp and pulled out from the vagina by tumbling it backwards. Bilateral round ligaments, uteroovarian ligaments, and bilateral fallopian tubes were clamped, cut, and doubly ligated. McCall culdoplasty was performed with high peritonization. The vaginal cuff was closed in a continuous fashion with 2/0 polyglactin suture. The vaginal hysterectomy was thereby completed. A linear incision was performed on the anterior vaginal mucosa, on about 2.5 cm below the external urethral opening, and the space was dissected until reaching the bladder base. Vesicovaginal ligaments were retracted laterally. Proximal part of the mesh was passed over arcus tendineus fascia pelvis (ATFP) with an obturator fossa guide. The posterior part of the four-arm mesh was passed through the obturator foramen (Figure 2). The anterior arms were placed as a midurethral sling as in the intravaginal slingplasty (IVS) procedure, supporting the midurethra and bladder (Figure 3). Posterior fringes of the mesh were fixed on sacrouterine and cardinal ligaments. About an 8–10 cm piece of monofilament, inelastic polypropylene tape is attached to the underside of the vaginal apex. Polypropylene sutures are placed into both of the sacrospinous and cardinal ligaments and threaded through the lateral edges of the apical sling and tied down, restoring apical support. Posterior mesh arms were fixed at the skin level by performing traction, but anterior part was not fixed at the midurethra and skin level, and tension free placement was performed.

Vagina mucosa was sutured in a continuous locking fashion with 2/0 polyglactin suture. In the same session, rectocele repair was performed. Rectovaginal fascial dissection was performed from posterior vagina and grade IV posterior prolapse was dissected broadly up to the apical region. Fascial defect was repaired with 2/0 polyglactin suture and a purse suture was placed. Excess vaginal mucosa was excised. A horizontal incision was made on the perineum, and perineoplasty was performed by enhancing puborectalis muscle medial region fixation. The vaginal mucosa and perineum were repaired using 2/0 polyglactin suture. Hemostasis was confirmed and the operation was concluded by placing a gauze tampon in the vagina. There were no intraoperative or immediate postoperative complications.

The patient was discharged home at the second postoperative day. Follow-up visits at the first, 3rd, and 6th months were normal. There was complete symptomatic relief and objective cure of the POP and urinary incontinence symptoms.

## 3. Discussion

Urinary incontinence and pelvic organ prolapse are common diseases that affect almost 1/3 of premenopausal women and approximately 45% of postmenopausal women.

International Continence Society (ICS) defines urinary incontinence (UI) as a condition where objectively demonstrated involuntary loss of urine is present, and this condition causes a social or hygienic problem [3, 4]. The most frequently encountered type is stress urinary incontinence (SUI), a symptom that refers to leakage of urine in case of increased abdominal pressure such as sneezing or coughing. SUI occurs when the intravesical pressure exceeds the maximum urethral pressure in the absence of detrusor activity. Pelvic organ prolapse (POP) is defined as the prolapse of the pelvic organs toward or through the vaginal opening, as a result of the weakening of the combination of nerves, muscles, and fascia, which normally sustain the anatomical position of pelvic organs [1, 2]. In women with SUI, nearly 60% also have POP [5]. Although POP is frequently asymptomatic, it may be associated with complaints such as pelvic pain, vaginal bleeding, and gastrointestinal or urinary symptoms [6, 7]. In many of these women, surgery is the only effective treatment option. Unfortunately, about 1 in 3 women need another surgical intervention after these operations [8]. Problems that occur in prolapsus can seriously affect the quality of life. Problems that can be seen together with POP include difficulty in walking, sitting, and standing up, backache, difficulty in urination, urinary retention, constipation, dyspareunia due to vaginal erosion, and sexual dysfunction [9].

POP and urinary incontinence are both common complaints and may frequently coexist in the same patient. Of all the women with SUI, 63% also have prolapse and 62% of those with prolapse have concomitant SUI [10]. Rosenzweig et al. [11] found that 60% of the women who had severe pelvic organ prolapse but did not have prominent urinary incontinence symptoms had latent urinary incontinence on urodynamic examination. Grady et al. found that 30% of the women who have cystocele also have abnormal bladder muscle contractions [12].

Anterior vaginal prolapse is defined as descent of the anterior vagina so that the urethral-vesical junction or any anterior point proximal to this is less than 3 cm above the plane of the hymen. These defects are not due to the strain and tension defects in the vaginal fascia only. Specific defects that occur in the supportive structures of the anterior vaginal wall also can cause anterior wall defects. Anterior wall defects are common and recurrences mostly occur in this region after POP operations. The recurrence rate after colporrhaphy anterior operation is reported to be between 20 and 40% [13]. This high recurrence rate indicates that classical plication of the anterior wall fascia is insufficient alone. In case specific defects in vaginal support structures go unnoticed, they can cause an increase in recurrence rates following traditional operations. Paravaginal defect repair was defined by Richardson in 1976. Paravaginal defect repair has been found to be important in strengthening the support structures of the anterior vaginal wall and protecting the normal anatomical position of the anterior wall. However, this repair is difficult to implement in routine practice and may have high complication rates. Therefore, repair operations utilizing polypropylene mesh have been proposed as a more effective treatment option rather than paravaginal repair and anterior colporrhaphy [14]. Numerous studies have evaluated and confirmed the effectiveness of meshes in POP surgery [15–17]. Mesh practices were first used vaginally by Julian; and recurrence rates and complications were found to be acceptably low [18].

*In conclusion*, in appropriately selected cases, we believe that anterior four-arm mesh implant and large posterior repair should be considered after vaginal hysterectomy. It is predicted that, when compared to the existing conventional methods, mesh surgery for the treatment of POP may decrease the recurrence rate after POP surgery. Future studies are needed to evaluate the utility of this technique for treatment of POP.
